# Impulsive actions and choices in laboratory animals and humans: effects of high *vs*. low dopamine states produced by systemic treatments given to neurologically intact subjects

**DOI:** 10.3389/fnbeh.2014.00432

**Published:** 2014-12-23

**Authors:** Valérie D’Amour-Horvat, Marco Leyton

**Affiliations:** ^1^Department of Psychology, McGill UniversityMontreal, QC, Canada; ^2^Department of Psychiatry, McGill UniversityMontreal, QC, Canada; ^3^Center for Studies in Behavioral Neurobiology, Concordia UniversityMontreal, QC, Canada

**Keywords:** impulsivity, compulsivity, addiction, gambling, motivation

## Abstract

Increases and decreases in dopamine (DA) transmission have both been suggested to influence reward-related impulse-control. The present literature review suggests that, in laboratory animals, the systemic administration of DA augmenters preferentially increases susceptibility to premature responding; with continued DA transmission, reward approach behaviors are sustained. Decreases in DA transmission, in comparison, diminish the appeal of distal and difficult to obtain rewards, thereby increasing susceptibility to temporal discounting and other forms of impulsive choice. The evidence available in humans is not incompatible with this model but is less extensive.

## Overview

Both low and high dopamine (DA) states have been proposed to affect impulse-control. These contrasting views might reflect different roles in different types of impulsivity. To address this possibility, we conducted literature searches using PubMed and Google Scholar, effective to October 20, 2014. The following terms were entered: impulsive action, stop signal, go/no-go, premature respon*, five-choice serial reaction time task (5-CSRT), differential reinforcement of low rates (DRL) of responding, simple reaction time task, conditional reaction time, impulsive choice, delay discount*, temporal discount*, effort discount*, probability discount*, Iowa Gambling, gambling, and decision-making, each entered separately in combination with dopamine, dopamine agonist, and dopamine antagonist. Papers were then selected for suitability with a focus on studies conducted with neurologically intact subjects following acute pharmacological challenges administered systemically. Although this focus ignores regionally specific effects within the brain, it identifies research with greater immediate clinical relevance with respect to the routes of drug and medication administration in humans. The strategy was supplemented by additional publications noted in the reference lists, and yielded a total of 88 papers. Overall, the reviewed evidence suggests that, in laboratory rodents, elevated DA transmission increases susceptibility to premature responding, while low DA increases preference for immediately available rewards over larger but more distal or difficult to attain ones. The evidence in neurologically intact humans is not incompatible with this view but is less extensive.

## Impulsivity subtypes

Impulse-control is a multifaceted construct (Solanto et al., 2001; Brewer and Potenza, 2008). One common demarcation separates (1) impulsive choice, defined as “actions initiated without due deliberation of other possible options or outcomes”; and (2) impulsive action, defined as behaviors that are “premature, mistimed, difficult to suppress and control” (Dalley et al., 2008). Impulsive action, in turn, might be subdivided into (a) premature responding; and (b) the inability to inhibit an initiated response. These subtypes are based on behavioral and cognitive requirements to perform the tasks used in research experiments (Winstanley et al., 2006), and disturbances are seen in a wide range of psychiatric disorders, such as attention deficit/hyperactivity disorder, pathological gambling and substance use disorders (American Psychiatric Association, 2013). There is compelling evidence that DA plays an important, if not fully understood, role in each of these disorders, and, moreover, of a causal relationship between DA agonist medication and the onset of various impulse-control problems such as pathological gambling, hypersexuality, compulsive shopping, compulsive drug-seeking, and binge eating (Gallagher et al., 2007; Dagher and Robbins, 2009; Moore et al., 2014).

In laboratory animals impulsive action has been assessed most commonly with the stop-signal task (SST; Logan et al., 1984), the go/no-go task (de Wit et al., 2002), the 5-CSRT; Robbins, 2002, the DRL task (e.g., Seiden et al., 1979), and the simple reaction time task (Amalric and Koob, 1987). Versions of all five tasks have been used in laboratory rodents and humans, but the 5-CSRT literatures consist predominantly of animal studies (Winstanley, 2011). While all tasks can measure premature responding, the SST and go/no-go tasks were also designed to measure the ability to inhibit a motor response. The go/no-go task measures the ability to inhibit responses to inappropriate cues, whereas the SST measures the speed at which an already initiated response can be inhibited (Eagle and Baunez, 2010). The 5-CSRT, DRL and simple reaction time tasks involve “waiting” before making a response to obtain a reinforcer (see Box 1 for a more detailed description of these tasks). Impulsive choice, in comparison, reflects the preference for immediately available small rewards over larger but more distal ones, and is commonly evaluated with temporal discounting procedures such as the delay discounting task (DDT; Ainslie, 1975), effort discounting paradigms (e.g., Floresco et al., 2008), probabilistic discounting tasks (e.g., St Onge and Floresco, 2009) and gambling-like tasks, such as the Iowa Gambling Task (IGT; Bechara et al., 1994; see Box 2 for more detailed descriptions of these tasks).

Box 1 **Measures of impulsive action.**

1. ***The five-choice serial reaction time task* (5-CSRT; Robbins, 2002)**

A visual stimulus is presented at one of five locations, and responding must be withheld until the stimulus signals that responding is appropriate. Impulsive behavior is measured by the number of responses made before the onset of the stimulus.

2. ***The differential reinforcement of low rates (DRL) of responding task* (e.g., Seiden et al., 1979)**

To obtain a reinforcer, subjects are required to withhold from responding for a fixed period of time and then to respond. Delays during which subjects are required to withhold from responding typically range from 10 s (DRL 10) to 72 s (DRL 72). Premature responses on this task consist of those made before this period of time has elapsed. Such responses reset the trial and are not reinforced. In some instances, subjects are required to respond after a fixed period of time has elapsed, but the response must occur within a certain delay (e.g., DRL 10–14), otherwise late responses are not reinforced.

3. ***The simple reaction time task* (Amalric and Koob, 1987)**

Each trial begins when subjects press on a lever. They must hold the lever down for a variable period of time, until a visual or auditory stimulus is presented. Following the presentation of this stimulus, subjects must release the lever within a pre-determined delay. Incorrect trials consist of those during which the lever was released prior to the stimulus onset (i.e., anticipated or premature responses) or after the delay has elapsed following the stimulus onset (i.e., delayed responses).

4. ***The stop-signal task* (SST; Logan et al., 1984)**

Subjects initiate a motor response following a go signal, and reaction times (RT) are determined. On a small proportion of trials, a stop signal follows the go signal. Sometimes the stop signal appears well before the subject’s RT limit, thereby providing sufficient time to inhibit the response. On other trials though the stop signal occurs very close to when the subject would normally respond, providing little time to inhibit the behavior. The longer the interval required to inhibit responses, the longer the stop signal response time (SSRT), and the poorer the inhibitory control.

5. ***The go/no-go task* (de Wit et al., 2002)**

Only one signal is presented per trial. The go signal is much more frequent than the no-go signal, thus priming subjects to initiate a motor response. On this task, poor inhibitory control is quantified by the number of responses on no-go trials (i.e., errors of commission).

Box 2 **Measures of impulsive choice.**

1. ***The delay discounting task* (DDT; Ainslie, 1975)**

Subjects must choose between a small reinforcer delivered immediately and a large reinforcer delivered after a given delay. On separate trials, the value of the immediate reinforcer or the delay to obtain the large reinforcer is changed, until subjects reach a point where both options are chosen equally. This point is called the indifference point. This step is repeated several times to obtain multiple points, which yield a hyperbolic discounting curve. The measure of interest is the steepness of that curve, which is called the discounting rate, with greater steepness indicating greater discounting of delayed reinforcers (i.e., impulsive choice). Note that in studies conducted in human subjects, reinforcers can be hypothetical or real.

2. ***Effort discounting tasks* (e.g., Floresco et al., 2008)**

Subjects must choose between a small reinforcer that can be easily obtained and a large reinforcer that requires greater effort to obtain. On separate trials, the value of the easy reinforcer or the amount of effort required to obtain the large reinforcer is changed, until subjects reach a point where both options are chosen equally. This point is called the indifference point. This step is repeated several times to obtain multiple points, which yield a hyperbolic discounting curve. The measure of interest is the steepness of that curve, which is called the discounting rate, with greater steepness indicating greater effort discounting, meaning that subjects are less willing to exert effort to obtain the larger reinforcer (i.e., impulsive choice).

3. ***Probability discounting tasks* (e.g., St Onge and Floresco, 2009)**

Subjects must choose between a small reinforcer that is delivered with greater certainty, and a larger, more uncertain reinforcer that is delivered according to various probabilities. On separate trials, the value of the small reinforcer or the probability at which the large reinforcer is delivered is changed, until subjects reach a point where both options are chosen equally. This point is called the indifference point. This step is repeated several times to obtain multiple points, which yield a hyperbolic discounting curve. The measure of interest is the steepness of that curve, which is called the discounting rate, with greater steepness indicating greater probability discounting, meaning that subjects choose the large reinforcer despite low probabilities of its delivery (i.e., impulsive choice).

4. ***The Iowa Gambling Task* (IGT; Bechara et al., 1994)**

Subjects are asked to pick cards from four decks, two of which result in overall gain due to frequent small gains and infrequent small losses, and the remaining two decks resulting in overall loss due to frequent large gains but infrequent larger losses. As subjects are not explicitly told about these contingencies, they must learn the patterns of wins and losses associated with each deck, and guide their choice toward the advantageous decks (i.e., those providing smaller immediate rewards in prospect of avoiding a long-term loss) to obtain a positive outcome. Therefore, the IGT requires learning for optimal performance (Fellows and Farah, 2005).

While multiple other neurotransmitters also influence performance on these tasks (Winstanley et al., 2003; Winstanley, 2011), the focus of the present review is on the role of DA, both in laboratory animals and in healthy human subjects. With a few noted exceptions, we will specifically review studies on the effects of acutely administered drugs given systemically.

## Dopaminergic manipulations and impulsive behavior

### Impulsive action (premature responding; inability to stop an initiated behavior)

#### Animal studies

In laboratory rodents, a fairly consistent finding has been that DA augmenting drugs increase premature responding (see Table 1). This effect has been observed on the 5-CSRT following the acute administration of amphetamine (0.15–1.6 mg/kg, i.p.; Cole and Robbins, 1987; Harrison et al., 1997; van Gaalen et al., 2006b, 2009; Pattij et al., 2007; Loos et al., 2010; Fletcher et al., 2011; Baarendse and Vanderschuren, 2012; see also Zeeb et al., 2009), methylphenidate (2.0, 2.5, 5.0 mg/kg, i.p.; Navarra et al., 2008; Milstein et al., 2010), cocaine (5–20 mg/kg, i.p.; van Gaalen et al., 2006b; Winstanley et al., 2007; Fletcher et al., 2011), and the selective DA reuptake inhibitor, GBR 12909 (1.0, 2.5, 5.0, 10.0 mg/kg, i.p.; van Gaalen et al., 2006b; Loos et al., 2010; Baarendse and Vanderschuren, 2012; Fernando et al., 2012). Two studies reported a null effect for methylphenidate (0.3–4.0 mg/kg, i.p.) on premature responding, although a nonsignificant trend of a dose-dependent increase was found (Fernando et al., 2012; Paterson et al., 2012). Orally administered modafinil (32, 64, 128 mg/kg) failed to increase impulsive action on the standard 5-CSRT, but did so at the two highest doses on a modified version with shorter stimulus duration and lower stimulus intensity (Waters et al., 2005). On the DRL, increased premature responding has been reported for schedules varying from 10 to 72 s following acute administration of amphetamine (0.5 mg/kg, s.c.; 0.3–4.0 mg/kg, i.p.; Sanger et al., 1974; Canon and Lippa, 1977; Seiden et al., 1979; Britton and Koob, 1989; Wenger and Wright, 1990; van Hest et al., 1992; Richards et al., 1993; Sabol et al., 1995; Bizot, 1998; Wiley et al., 2000; Ferguson et al., 2001; Liao and Cheng, 2005; Cheng and Liao, 2007), methylphenidate (2.0–20.0 mg/kg, i.p.; Seiden et al., 1979; Ferguson et al., 2001), cocaine (1, 2, 4 mg/kg, i.v.; 10, 20, 40 mg/kg, p.o.; 3–16 mg/kg, i.p.; Woolverton et al., 1978; Wenger and Wright, 1990; Ma et al., 1999; Wang et al., 2001; Cheng et al., 2006), and modafinil (64, 128 mg/kg, i.p.; Bizot, 1998). On simple reaction time tasks, increased premature responding has been observed in response to amphetamine administration (0.8 mg/kg, s.c.; 1.0 mg/kg, i.p.; Baunez et al., 1995; Blokland et al., 2005). Approach toward the reward persists with sustained increases in DA transmission (Salamone et al., 1991, 2001; Robinson and Berridge, 2008; Howe et al., 2013; Leyton and Vezina, 2014).

**Table 1:** **Effects of increased DA transmission on impulsive action in laboratory animals**.

Drug	Dose (mg/kg)	Study	Species tested	5-CSRT	DRL	SRT	SST	Go/No-Go	Comments
AMPH	0.25, 0.5, 1	Baarendse and Vanderschuren (2012)	Male Lister-hooded rats	↑					All doses increased premature responses
	0.6, 0.8 (s.c.)	Baunez et al. (1995)	Male Wistar rats			↑			Dose-dependent decrease in number of correct trials; increased number of premature responses at highest dose only
	0.5, 1	Bizot (1998)	Male Wistar rats		↑				Both doses increased response rate, but only 1 mg/kg increased number of premature responses and decreased reinforcement rate
	0.3, 1	Blokland et al. (2005)	Male Lewis rats			↑			Increased number of premature responses and of mean RT at highest dose only
	0.5 (s.c.)	Britton and Koob (1989)	Male Wistar rats		↑				Increased number of premature responses and decreased reinforcement rate; DRL 60
	0.5, 1	Canon and Lippa (1977)	Male Wistar rats		↑				Decrease in reinforcement rate at both doses; DRL 10
	1	Cheng and Liao (2007)	Male Sprague Dawley rats		↑				Decreased number of reinforced responses and increased number of premature responses; DRL 10
	0.4, 0.8, 1.6	Cole and Robbins (1987)	Male hooded rats	↑					Dose-dependent increase in premature responses
	0.3, 1	Eagle et al. (2009)	Male Lister-hooded rats				↑		Increased stop signal response time (SSRT) at 1.0 mg/kg (nonsignificant decrease in SSRT at 0.3 mg/kg); decreased go and stop accuracy at both doses
	0.3, 1	Eagle and Robbins (2003)	Male Lister-hooded rats				–		No effect
	0.125, 0.25, 0.5, 1	Feola et al. (2000)	Sprague Dawley rats				↓ (slow) – (fast)		Reduced SSRT at 0.5 mg/kg in slow stoppers but not in fast stoppers
	0.1, 0.3, 0.65, 1	Ferguson et al. (2001)	Sprague Dawley rats		↑				Increased response rate at all doses but 0.1 mg/kg; dose dependent increase in very premature responses; DRL 10–14
	0.15, 0.3, 0.6	Fletcher et al. (2011)	Male Long Evans rats	↑					All doses increased premature responses
	0.2, 0.4, 0.8	Harrison et al. (1997)	Male Lister-hooded rats	↑					Increased premature responding at 0.4 and 0.8 mg/kg
	0.5, 1	Liao and Cheng (2005)	Male Wistar rats		↑				Both doses increased number of premature responses and decreased reinforcement rate; DRL 10
	5-CSRT: 0.25, 0.5, 1	Loos et al. (2010)	Male C57BL/6J mice	↑				–	Increased premature responding at 1.0 mg/kg
	Go/No-Go: 0.5, 1		Male DBA/2J mice	–				–	
	0.5	Pattij et al. (2007)	Male Wistar rats	↑				
	0.5, 1, 2	Richards et al. (1993)	Male Sprague Dawley rats		↑				All doses increased response rate and decreased reinforcement rate; DRL 72
	0.25, 0.5, 1, 2, 4	Sabol et al. (1995)	Male Sprague Dawley rats		↑				Dose-dependent increase in number of premature responses and decrease in reinforcement rate; DRL 36
	0.5, 1, 2, 4	Sanger et al. (1974)	Male Hooded rats		↑				Dose-dependent increase in number of premature responses and decrease in reinforcement rate; DRL 15
	0.375, 0.75, 1.5, 3	Seiden et al. (1979)	Male Sprague Dawley rats		↑				Dose-dependent increase in number of premature responses and decrease in reinforcement rate
	0.2, 0.5, 1	van Gaalen et al. (2006b)	Male Wistar rats	↑					Increased premature responding at 0.5 and 1.0 mg/kg
	0.3, 0.6, 1.2	van Gaalen et al. (2009)	Male Wistar rats	↑					Increased premature responding at 0.6 and 1.2 mg/kg (Two-choice serial reaction time task)
	0.5, 1, 2, 4	van Hest et al. (1992)	Male Wistar rats		↑				Dose-dependent increase in response rate up to 2.0 mg/kg; DRL 72
	0.1, 0.3, 1, 2, 3	Wenger and Wright (1990)	Male CD rats		↑				Increased number of premature responses at doses of 1–3 mg/kg; DRL 10–14
	0.1, 0.3, 1, 3	Wiley et al. (2000)	Long-Evans hooded rats		↑				Two highest doses decreased number of reinforcers earned; DRL 15
MPH	0.3, 1	Eagle et al. (2007)	Male Lister-hooded rats				↓ (slow) ↑ (fast)		Decreased SSRT in rats with initially slow SSRT (0.3 mg/kg) but increased SSRT in rats with initially fast SSRTs at both doses
	2, 3.25, 4.5, 7.5	Ferguson et al. (2001)	Sprague Dawley rats		↑				All doses increased response rate; dose dependent increase in very premature responses; DRL 10–14
	0.3, 1, 3	Fernando et al. (2012)	Male Lister-hooded rats	–					Non-significant trend of dose dependent increase in premature responses
	2	Milstein et al. (2010)	Male Lister-hooded rats	↑					All doses increased premature responding
	2.5, 5	Navarra et al. (2008)	Male Long Evans rats	↑					All doses increased premature responding
	0.5, 1, 2, 4	Paterson et al. (2012)	Male Long Evans rats	–				
	2.5, 5, 10, 20	Seiden et al. (1979)	Male Sprague Dawley rats		↑				Increased number of premature responses and decreased reinforcement rate at 10 and 20 mg/kg; DRL 17.5
Cocaine	15	Cheng et al. (2006)	Male Sprague Dawley rats		↑				Increased number of premature responses; DRL 12
	1, 2, 4 (i.v.) 10, 20, 40 (p.o.)	Ma et al. (1999)	Male Sprague Dawley rats		↑				Dose-dependent increase in number of premature responses and decrease in reinforcement rate; DRL 45
	7.5, 15, 30	Fletcher et al. (2011)	Male Long Evans rats	↑					Increased premature responses at 15 mg/kg only
	5, 10, 15	Paine and Olmstead (2004)	Male Long Evans rats					↑	Increased number of responses made during no-go trials at 15 mg/kg
	5, 10, 20	van Gaalen et al. (2006b)	Male Wistar rats	↑					All doses increased premature responding
	1, 2, 4 (i.v.) 10, 20, 40 (p.o.)	Wang et al. (2001)	Male Sprague Dawley rats		↑				Dose-dependent increase in number of premature responses and decrease in reinforcement rate; DRL 45
	0.1, 0.3, 1, 3, 5.6, 10	Wenger and Wright (1990)	Male CD rats		↑				Increased number of premature responses at doses of 3–10 mg/kg; DRL 10–14
	5, 10, 20	Winstanley et al. (2007)	Male Sprague Dawley rats	↑					All doses increased premature responding
	4, 8, 16, 32	Woolverton et al. (1978)	Male Sprague Dawley rats		↑				Increased number of premature responses and decreased reinforcement rate at 8 and 16 mg/kg; DRL 20
Modafinil	16, 32, 64, 128	Bizot (1998)	Male Wistar rats		↑				Increased number of premature responses and decreased reinforcement rate at 64 and 128 mg/kg. Highest dose also increased response rate
	3, 10, 30	Eagle et al. (2007)	Male Lister-hooded rats				↓ (slow) – (fast)		Decreased SSRT in rats with initially slow SSRT but no effect in rats with initially fast SSRTs at 10 mg/kg
	32, 64, 128 (p.o.)	Waters et al. (2005)	Male Lister-hooded rats	– (std) ↑ (load)					No effect on standard task but increased premature responses on modified version with shorter stimulus duration and reduced stimulus intensity at 64 and 128 mg/kg
GBR 12909	2.5, 5, 10	Baarendse and Vanderschuren (2012)	Male Lister-hooded rats	↑					Increased premature responding at 10 mg/kg only
	0.1, 0.3, 1, 3 (s.c.) 5, 10 (i.p.)	Bari et al. (2009)	Male Lister-hooded rats				–		No effect at 0.1–3.0 mg/kg; Decreased stop accuracy at 5–10 mg/kg
	0.5, 1, 2.5, 5	Fernando et al. (2012)	Male Lister-hooded rats	↑					All doses increased premature responding except for lowest dose
	2.5, 5, 10	Loos et al. (2010)	Male C57BL/6J mice	↑					Increased premature responding at 5 and 10 mg/kg
			Male DBA/2J mice	–					Increased premature responding at 10 mg/kg only

*↑ = increase in impulsivity, ↓ = decrease in impulsivity, – = no effect. Doses are expressed in mg/kg. Route of administration is i.p., unless otherwise specified. AMPH =amphetamine, MPH = methylphenidate*.

*Premature responding = Five-choice serial reaction time task (5-CSRT), Differential reinforcement of low rates of responding task (DRL); Simple reaction time task (SRT); Inability to inhibit prepotent responses = Stop-signal task (SST), Go/No-Go task*.

Dopamine antagonists have the opposite effects of agonists. Decreased premature responding has been observed on the 5-CSRT following acute administration of the DA D1 receptor antagonist, SCH 23390 (0.05, 0.075 mg/kg, i.p. and 10–30 µg/kg, i.p.; Harrison et al., 1997; van Gaalen et al., 2006b), while D2 antagonists, such as sulpiride (40, 60 mg/kg, i.p.) and eticlopride (0.06–0.1 mg/kg, i.p.), increase the latency to initiate a response, thus reducing the propensity to impulsive responding (Harrison et al., 1997; van Gaalen et al., 2006b; see Table 2). This apparent differential contribution of D1 and D2 receptors to impulsive responding might reflect their proposed roles in approach behaviors more generally, with D1 receptor stimulation thought to induce behavioral activation and heighten the signal-to-noise salience of appetitive cues while D2 stimulation releases the “brake” on behavior (Frank, 2005; Tai et al., 2012). Importantly, re-engaging this hypothesized “brake” with eticlopride also diminishes the ability of DA augmenters to increase premature responding (van Gaalen et al., 2006b). On the DRL, the number of premature responses was diminished by acute doses of SCH 23390 (0.02–0.1 mg/kg, i.p.) and the D2 antagonists raclopride (0.2–0.5 mg/kg, i.p.; Liao and Cheng, 2005; Cheng and Liao, 2007) and haloperidol (0.25, 0.5 µg/kg, s.c.; 0.16, 0.32 mg/kg, i.p.; Britton and Koob, 1989; van Hest et al., 1992). On simple reaction time tasks, several effects have been reported following acute treatment with DA antagonists. Decreased premature responses have been observed in response to SCH 23390 (0.025–0.2 mg/kg, i.p.), raclopride (0.05–0.8 mg/kg, i.p.) and haloperidol (0.05–0.4 mg/kg, i.p.; Marrow et al., 1993). Dopamine antagonists can also impair performance by lengthening reaction times (RT), resulting in a greater number of delayed responses while leaving premature responding intact. These effects have been seen following administration of eticlopride (0.01, 0.02 mg/kg, s.c.; Smith et al., 2000), flupenthixol (0.2, 0.4 mg/kg, i.p.; Amalric and Koob, 1987), and raclopride (50–200 µg/kg, s.c.; 0.05 mg/kg, s.c.; 0.8 mg/kg, i.p.; Amalric et al., 1993; Marrow et al., 1993; Baunez et al., 1994, 1995). However, null effects have also been reported in response to eticlopride (0.01, 0.03 mg/kg, i.p.; Blokland et al., 2005), SCH 23390 (5, 10, 20 µg/kg, s.c.; Amalric et al., 1993) and flupenthixol (0.05, 0.1, 0.2, 0.4 mg/kg, i.p.; Marrow et al., 1993). These slightly discrepant results might reflect differences in the specifics of the task, such as the delay after the onset of the stimulus, the behavior performed (e.g., nose poke *vs*. lever press), or the effect of these compounds on motivation. For example, Blokland et al. (2005) found that the highest dose of eticlopride administered in their study (0.03 mg/kg, i.p.) decreased motivation to obtain food, as assessed by a progressive-ratio schedule.

**Table 2:** **Effects of decreased DA transmission on impulsive action in laboratory animals**.

Drug	Dose (mg/kg)	Study	Species tested	5-CSRT	DRL	SRT	SST	Comments
Haloperidol	25, 50 µg/kg (s.c.)	Britton and Koob (1989)	Male Wistar rats		↓			Both doses decreased number of premature responses and increased reinforcement rate; DRL 60
	0.05, 0.1, 0.2, 0.4	Marrow et al. (1993)	Male Sprague Dawley rats			↓		Dose-dependent decrease in number of premature responses
	0.02, 0.04, 0.08, 0.16, 0.32	van Hest et al. (1992)	Male Wistar rats		↓			Decreased response rate at 0.16 and 0.32 mg/kg; DRL 72
Raclopride	50, 100, 200 µg/kg (s.c.)	Amalric et al. (1993)	Male Wistar rats			↓		Dose-dependent decrease in number of correct trials; all doses increased number of delayed responses
	0.05 (s.c.)	Baunez et al. (1994)	Male Wistar rats			↓		Increased number of delayed responses
	0.05 (s.c.)	Baunez et al. (1995)	Male Wistar rats			↓		Increased number of delayed responses
	0.2, 0.5	Cheng and Liao (2007)	Male Sprague Dawley rats		↓			Both doses decreased response rate, but only the highest dose decreased the number of reinforced responses; DRL 10
	0.2, 0.4	Liao and Cheng (2005)	Male Wistar rats		↓			Both doses decreased number of premature responses and highest dose decreased reinforcement rate; DRL 10
	0.05, 0.1, 0.2, 0.4, 0.8	Marrow et al. (1993)	Male Sprague Dawley rats			↓		Dose-dependent decrease in number of correct trials and number of premature responses, and dose-dependent increase in RT; delayed responses increased at highest dose
Flupenthixol	0.1, 0.2, 0.4	Amalric and Koob (1987)	Male Wistar rats			↓		Reduced number of correct trials by increasing number of delayed responses at 0.2 and 0.4 mg/kg; increased RT during correct trials and decreased overall responding at highest dose
	0.01, 0.04, 0.125	Eagle et al. (2007)	Male Lister-hooded rats				–	No effect on SSRT in either slow or fast stoppers
	0.05, 0.1, 0.2, 0.4	Marrow et al. (1993)	Male Sprague Dawley rats			–		Dose-dependent decrease in number of correct trials at 0.1–0.4 mg/kg
SCH 23390	5, 10, 20 µg/kg (s.c.)	Amalric et al. (1993)	Male Wistar rats			–	
	0.02, 0.05	Cheng and Liao (2007)	Male Sprague Dawley rats		↓			Both doses decreased number of premature responses; DRL 10
	0.025, 0.05, 0.075	Harrison et al. (1997)	Male Lister-hooded rats (5-HT depleted)	↓				Decreased number of premature responses at 0.05 and 0.075 mg/kg
	0.05, 0.1	Liao and Cheng (2005)	Male Wistar rats		↓			Both doses decreased number of premature responses; DRL 10
	0.025, 0.05, 0.1, 0.2	Marrow et al. (1993)	Male Sprague Dawley rats			↓		Dose-dependent decrease in number of correct trials and number of premature responses
	5, 10, 20, 30 µg/kg	van Gaalen et al. (2006b)	Male Wistar rats	↓				Decreased premature responding at 10, 20 and 30 µg/kg
Sulpiride	20, 40, 60	Harrison et al. (1997)	Male Lister-hooded rats	–				No effect on number of premature responses, but increased response latency at 40 and 60 mg/kg
Eticlopride	0.01, 0.03	Blokland et al. (2005)	Male Lewis rats			–		No effect at any dose on premature responses, but highest dose decreased food motivation on PR10 schedule;
	0.005, 0.01, 0.02 (s.c.)	Smith et al. (2000)	Male Wistar rats			↓		Dose-dependent decrease in number of correct trials due to increase in delayed responses at 0.01 and 0.02 mg/kg; increased RT at 0.02 mg/kg
	0.06, 0.08, 0.1	van Gaalen et al. (2006b)	Male Wistar rats	–				No effect on number of premature responses, but increased response latency at all doses and reduced ability of amphetamine, cocaine and nicotine to increase premature responses

*↑ = increase in impulsivity, ↓ = decrease in impulsivity, – = no effect. Doses are expressed in mg/kg, unless otherwise specified. Route of administration is i.p., unless otherwise specified*.

*Premature responding = Five-choice serial reaction time task (5-CSRT), Differential reinforcement of low rates of responding task (DRL); Simple reaction time task (SRT); Inability to inhibit prepotent responses = Stop-signal task (SST)*.

In studies using the SST and go/no-go tasks, the results have been similar. An acute dose of GBR 12909 (5, 10 mg/kg, i.p.) accelerated both go and stop SST RT leading to an increased number of premature responses (Bari et al., 2009). Although low to moderate doses of amphetamine (0.5, 1.0 mg/kg, i.p. in mice; Loos et al., 2010) and cocaine (5, 10 mg/kg, i.p. in rats; Paine and Olmstead, 2004) had no effect, a higher dose of cocaine (15 mg/kg, i.p.) increased premature responding, as indexed by increased commission errors (responding during no-go trials; Paine and Olmstead, 2004).

The ability to inhibit an initiated response is also altered by DAergic drugs but the pattern of effects differs compared to those seen on premature responding. In studies using the SST, the effects of amphetamine, methylphenidate and modafinil depended on whether the animals had slow or fast inhibitory responses under placebo. In rats with poor inhibitory control (slow responders), all three drugs improved performance by shortening the time required to inhibit an initiated response (Feola et al., 2000; Eagle and Robbins, 2003; Eagle et al., 2007, 2009). The opposite effect was observed in fast responders (0.3, 1.0 mg/kg modafinil, i.p.; Eagle et al., 2007). Administration of the D1/D2 receptor antagonist flupenthixol (0.01–0.125 mg/kg, i.p.), in comparison, had no effect on the ability to inhibit responses on the SST in either fast or slow responders (Eagle et al., 2007).

Together then, the weight of evidence from these studies in laboratory rodents indicates that elevations in DA transmission can have two main effects on impulsive actions: they increase premature responding while also improving the ability to inhibit prepotent responses in impulsive animals.

#### Human studies

To our knowledge, there are no studies of the effects of DA augmenters on premature responding in healthy humans. There is a small literature, though, describing effects on the ability to inhibit initiated responses (Table 3). In agreement with studies in laboratory animals, the acute administration of low-to-moderate doses of oral *d*-amphetamine (10–20 mg) had differential effects on SST performance depending on the subject’s baseline performance. In individuals with poor baseline inhibitory control (i.e., slow stoppers), *d*-amphetamine improved the ability to inhibit an initiated response, while having no effect in fast stoppers (de Wit et al., 2000, 2002). A similar pattern has been observed on the go/no-go task, where more impulsive subjects at baseline showed a reduction in the number of commission errors following both 10 and 20 mg of *d*-amphetamine (de Wit et al., 2002). These results are consistent with those seen in children and adults with attention-deficit/hyperactivity disorder, where the ability to inhibit responses is improved following the administration of oral methylphenidate (Tannock et al., 1989; Aron et al., 2003). They are also in accordance with a recent study by Aarts et al. (2014), in which individuals with greater DA synthesis capacity performed poorly when they anticipated a large reward on a modified Stroop task. The authors proposed that DA release in response to the large rewards may “overdose” an already DA-rich system, and have detrimental effects on performance. These detrimental effects would potentially emerge with higher doses of *d*-amphetamine in fast stoppers. In one study, methylphenidate administration (40 mg) did not affect healthy adults’ performance on the SST or on the go/no-go task, but it reduced intra-individual RT which is indicative of increased attention (Costa et al., 2013). The lack of effect on overall performance could be explained by the low rate of inhibition errors in this particular sample of participants.

**Table 3:** **Effects of altered DA transmission on impulsive action in healthy human subjects**.

Drug	Dose	Study	Subjects tested	SST	Go/No-Go	Comments
**Increased DA transmission**
Amphetamine	20 mg	Acheson and de Wit (2008)	Healthy adults	–	
	10, 20 mg	de Wit et al. (2000)	Healthy adults	↓ (slow) – (fast)		Both doses decreased SSRT in slow stoppers, but no effect in fast stoppers
	10, 20 mg	de Wit et al. (2002)	Healthy adults	↓ (slow) – (fast)	↓ (slow) – (fast)	At 20 mg only, decreased SSRT in slow stoppers, but no effect in fast stoppers; both doses decreased commission errors in more impulsive subjects, but not in the less impulsive ones
	7.5, 15 mg/kg	Fillmore et al. (2005)	Healthy adults	–		
Methylphenidate	40 mg	Costa et al. (2013)	Healthy men	–	–
Bupropion	150, 300 mg	Acheson and de Wit (2008)	Healthy adults	–	
Pramipexole	0.25, 0.50 mg	Hamidovic et al. (2008)	Healthy adults		–
Tyrosine	2.0 g	Colzato et al. (2014)	Healthy women	↓	
**Decreased DA transmission**
Tyrosine depletion	100 g BCAA with tyrosine	Leyton et al. (2007)	Healthy men	↑		Increased commission errors when correct responses are rewarded
	and phenylalanine withheld					
	90 g BCAA	Lythe et al. (2005)	Healthy adults	–	

*↑ = increase in impulsivity, ↓ = decrease in impulsivity, – = no effect. Route of administration is p.o. BCAA = branched chain amino acids. Inability to inhibit prepotent responses = Stop-signal task (SST), Go/No-Go task*.

Decreasing DA release, in comparison, using the acute phenylalanine/tyrosine depletion method, has been reported to increase go/no-go commission errors, particularly in response to reward cues (Leyton et al., 2007), and diminish the ability to supress incorrect impulses, as measured with a sensitive electromyography index (Ramdani et al., 2014). In the converse experiment, administration of the DA precursor, tyrosine (2.0 g, p.o.), improved SST performance by reducing the time required to inhibit initiated responses (Colzato et al., 2014). This effect of tyrosine might reflect greater cognitive control in the prefrontal cortex, as the same research group has found that tyrosine improves performance on a demanding condition of the *N*-Back task (Colzato et al., 2013) while tyrosine depletion tended to reduce *N*-Back performance in people carrying the low activity met allele of the gene encoding for the enzyme, catechol-*O*-methyltransferase (Kelm and Boettiger, 2013). These effects on the ability to inhibit prepotent responses, though, have not been observed consistently in other studies following tyrosine depletion (McLean et al., 2004; Lythe et al., 2005) or following administration of *d*-amphetamine (7.5–15 mg/kg, p.o.; Fillmore et al., 2005) or the DA agonist, pramipexole (0.25–0.5 mg, p.o.; Hamidovic et al., 2008). It remains to be tested whether these divergent findings reflect baseline differences in performance or the need for more sensitive measures (Ramdani et al., 2014).

### Impulsive choice (choosing small immediately available rewards over delayed or more difficult to attain large rewards)

#### Animal studies

In most studies, the ability to delay responding to receive a larger reward is disrupted by decreased DA transmission (Cardinal et al., 2000; Wade et al., 2000; Denk et al., 2005; van Gaalen et al., 2006a; Floresco et al., 2008; Koffarnus et al., 2011) and improved by modest increases (see Tables 4, 5). Beneficial effects have been observed following the administration of methylphenidate (1.0, 3.0 mg/kg, i.p.; van Gaalen et al., 2006a), cocaine (7.5, 15 mg/kg, i.p.; Winstanley et al., 2007), GBR 12909 (5 mg/kg, i.p.; van Gaalen et al., 2006a) and amphetamine (0.25–0.6 mg/kg, i.p.; Cardinal et al., 2000; Isles et al., 2003; Winstanley et al., 2003; Floresco et al., 2008). Following the administration of higher doses of amphetamine (0.5–2.3 mg/kg, i.p.; 1.0 mg/kg, s.c.), the reported results are more variable, and both disruptions (Charrier and Thiébot, 1996; Evenden and Ryan, 1996; Cardinal et al., 2000; Isles et al., 2003; Helms et al., 2006; Koffarnus et al., 2011; Maguire et al., 2014) and improvements have been described (Wade et al., 2000; Winstanley et al., 2003). A high dose of pramipexole (0.32 mg/kg, s.c.) increased preference for the smaller reinforcer (Koffarnus et al., 2011), whereas moderate to high doses of methylphenidate (1.0–4.0 mg/kg, i.p.) had the opposite effect (Paterson et al., 2012). Interestingly, the effects of amphetamine and methylphenidate seem to depend, at least in part, on the order of presentation of delay. Disruptions have been reported when animals are exposed to the various delays in descending order (Maguire et al., 2014; Tanno et al., 2014), whereas improvements (Tanno et al., 2014) or a null effect (Maguire et al., 2014) have been described when the delays are presented in ascending order. It has been proposed that amphetamine and methylphenidate might have induced perseverative responding early in the sessions when delays were presented in descending order, and that continued perseverative responding was responsible for the enhanced choice of the smaller, immediate reinforcer throughout the sessions (Tanno et al., 2014).

**Table 4:** **Effects of increased DA transmission on impulsive choice in laboratory animals**.

Drug	Dose (mg/kg)	Study	Species tested	DD	ED	PD	rGT	Comments
AMPH	0.25, 0.5, 1	Baarendse et al. (2013)	Male Lister-hooded rats				↓	All doses increased choice for smaller but more certain reward with lowest risk of punishment
	0.3, 1.0, 1.6	Cardinal et al. (2000)	Male Lister-hooded rats	↑ (no cue)				Increased impulsivity at 1.0 and 1.6 mg/kg (no cue); decreased impulsivity at 0.3 mg/kg (cue present)
				↓ (cue)				
	0.25, 0.5, 1	Charrier and Thiébot (1996)	Male Wistar rats	↑				Reduced choice for larger reinforcer at 0.5 mg/kg
	0.3, 0.6, 1	Cocker et al. (2012)	Male Long Evans rats		↓			Lowest dose made hard-working rats more willing to exert effort, but higher doses made them less willing to work for the larger reinforcer. These higher doses made “slacker” rats more willing to work for larger reinforcer. Based on cognitive, not motor effort
					↑			
	0.3, 1	Evenden and Ryan (1996)	Male Sprague Dawley rats	↑				Reduced choice for larger reinforcer at 1.0 mg/kg
	0.125, 0.25, 0.5	Floresco et al. (2008)	Male Long Evans rats	↓ (0.25)	↓ (0.125, 0.25)			Increased choice for larger, delayed reinforcer at 0.25 mg/kg; increased (0.125 and 0.25 mg/kg) and decreased (0.5 mg/kg) willingness to work to obtain reinforcer
					↑ (0.5)	
	0.4, 0.8, 1.2	Helms et al. (2006)	Male C57BL/6J mice	↑				Decreased choice for larger reinforcer at 0.8 and 1.2 mg/kg
			Male DBA/2J mice	↑				Decreased choice for larger reinforcer at 0.8 and 1.2 mg/kg
	0.4, 0.6, 0.8, 1	Isles et al. (2003)	Male F2	↓ (0.4, 0.6)			
			C57B1/6xCBA/CA	↑ (0.8, 1)			
	0.032, 0.1, 0.32, 1 (s.c.)	Koffarnus et al. (2011)	Male Sprague Dawley rats	↑				Decreased choice of large reward at 1 mg/kg
	0.32, 1, 1.78	Maguire et al. (2014)	Male Sprague Dawley rats	– (ascend) ↑ (descend)				½ of rats showed increased impulsivity when delays presented in ascending order, and the other ½ showed the opposite effect; decreased choice of larger reinforcer at 1 and 1.78 mg/kg when delays presented in descending order
	0.3, 1, 1.5	Mitchell et al. (2011)	Male Long Evans rats			↓		Dose-dependent decrease in choice of risky reinforcer; risk associated with large reinforcer is mild footshock
	0.33, 1, 1.5	Simon et al. (2009)	Male Long Evans rats			↓		Dose-dependent decrease in choice of large risky reinforcer; risk associated with large reinforcer is mild footshock
	1.5	Simon et al. (2011)	Male Long Evans rats			↓		Decrease in choice of large risky reinforcer; risk associated with large reinforcer is mild footshock
	0.125, 0.25, 0.5, 1.0	St Onge and Floresco (2009)	Male Long Evans rats			↑		All doses increased risky choice
	0.5	St Onge et al. (2010)	Male Long Evans rats			↑ (descend) ↓ (ascend)		Increased risky choice (descending prob); decreased risky choice (ascending prob)
	0.1, 0.32, 0.56, 1, 1.78	Tanno et al. (2014)	Male Sprague Dawley rats	↓ (ascend) ↑ (descend)				Dose-dependent increase in choice of large reinforcer when delays presented in ascending order, but opposite effect when delays presented in descending order
	1, 2.5	van Enkhuizen et al. (2013)	Male C57BL/6N mice				↓	Increased choice for smaller but more certain reward with lowest risk of punishment at highest dose
	0.2, 0.5, 1	van Gaalen et al. (2006a)	Male Wistar rats	↓				All doses increased choice for large reinforcer
	0.5, 1	Wade et al. (2000)	Sprague-Dawley rats	↓				Dose-dependent increase in indifference point; Sig increased choice for larger reinforcer at 1 mg/kg
	0.3, 1.0, 1.5, 2.3	Winstanley et al. (2003)	Male Lister-hooded rats	↓				Increased choice for larger reinforcer at 0.3 and 2.3 mg/kg
	0.3, 1.0, 1.5	Zeeb et al. (2009)	Male Long Evans rats				↓	Increased choice for smaller but more certain reward with lowest risk of punishment at 1.0 and 1.5 mg/kg
MPH	0.5, 1, 2, 4	Paterson et al. (2012)	Male Long Evans rats	↓				Increased choice for larger reinforcer at 1, 2 and 4 mg/kg
	1, 3.2, 10, 17.8	Tanno et al. (2014)	Male Sprague Dawley rats	↓ (ascend) ↑ (descend)				Dose-dependent increase in choice of large reinforcer when delays presented in ascending order, but opposite effect when delays presented in descending order
	0.3, 1, 3	van Gaalen et al. (2006a)	Male Wistar rats	↓				All doses increased choice for large reinforcer
Modafinil	16, 32, 64	van Enkhuizen et al. (2013)	Male C57BL/6N mice				–	Increased choice of larger but riskier reward during first half of session, relative to second half, at the highest dose
Cocaine	5, 10, 15	Simon et al. (2009)	Male Long Evans rats			–	
	0.3, 1, 3	van Gaalen et al. (2006a)	Male Wistar rats	↓				Increased choice for larger reinforcer at 5 mg/kg only
	7.5, 15	Winstanley et al. (2007)	Male Sprague Dawley rats	↓				All doses increased choice for large reinforcer
GBR 12909	2.5, 5, 10	Baarendse et al. (2013)	Male Lister-hooded rats				–
	1, 3.2, 10 (s.c.)	Koffarnus et al. (2011)	Male Sprague Dawley rats	–			
	9, 16, 28.5	van Enkhuizen et al. (2013)	Male C57BL/6N mice				–	Increased choice of larger but riskier reward during second half of session, relative to first half, at the lowest dose
	2, 5	van Gaalen et al. (2006a)	Male Wistar rats	↓				Increased choice for larger reinforcer at 5 mg/kg only
Pramipexole	0.032, 0.1, 0.32 (s.c.)	Koffarnus et al. (2011)	Male Sprague Dawley rats	↑				Decreased choice for larger reinforcer at highest dose only

*↑ = increase in impulsivity, ↓ = decrease in impulsivity, – = no effect. Doses are expressed in mg/kg. Route of administration is i.p., unless otherwise specified. MPH = methylphenidate, AMPH = amphetamine. Impulsive choice = Delay discounting (DD), Effort discounting (ED), Probability discounting (PD), and Rat Gambling Task (rGT)*.

**Table 5:** **Effects of decreased DA transmission on impulsive choice in laboratory animals**.

Drug	Dose (mg/kg)	Study	Species tested	DD	ED	PD	rGT	Comments
Haloperidol	0.2	Denk et al. (2005)	Male Lister-hooded rats	↑	↑			Impairment on both delay and effort discounting tasks
	0.01, 0.03 (s.c.)	Evenden and Ryan (1996)	Male Sprague-Dawley rats	– (standard)				No effect with standard delays, but increased choice of larger reinforcer with shorter delays
				↓ (short)				
	0.01, 0.032, 0.1 (s.c.)	Koffarnus et al. (2011)	Male Sprague Dawley rats	↑				Reduced choice for larger reinforcer at 0.1 mg/kg
	0.1	Salamone et al. (1991)	Male albino rats		↑			Decreased ability to exert effort to obtain reward
Raclopride	40, 80, 120 µg/kg	Wade et al. (2000)	Sprague-Dawley rats	↑				Decreased indifference point at 80 and 120 µg/kg
Flupenthixol	0.125, 0.25, 0.5	Cardinal et al. (2000)	Male Lister-hooded rats	↑				Increased choice of small reinforcer at 0.125 mg/kg in absence of cue, and at 0.25 mg/kg in presence of cues
	0.125, 0.25, 0.5	Floresco et al. (2008)	Male Long Evans rats	↑	↑			Increased choice of small reinforcer at 0.5 mg/kg; decreased willingness to work for reinforcers at 0.25 and 0.5 mg/kg
	0.4	St Onge et al. (2010)	Male Long Evans rats			↓		Decreased risky choice
	25, 50, 100 µg/kg	Wade et al. (2000)	Sprague-Dawley rats	↑				Decreased indifference point at 50 and 100 µg/kg
	0.125	Winstanley et al. (2003)	Male Lister-hooded rats	–			
	0.01, 0.02, 0.03	van Gaalen et al. (2006a)	Male Wistar rats	↑				Decreased ability to delay reward at 0.02 and 0.03 mg/kg
Eticlopride	0.01, 0.03, 0.05	Simon et al. (2011)	Male Long Evans rats			–		No effect at any dose; risk associated with large reinforcer is mild footshock
	0.01, 0.03	St Onge and Floresco (2009)	Male Long Evans rats			↓		Both doses decreased risky choice, but only the highest dose decreased it when probabilities were high
	0.03, 0.06, 0.09	van Gaalen et al. (2006a)	Male Wistar rats	–			
	0.01, 0.03, 0.06	Zeeb et al. (2009)	Male Long-Evans rats				↓	Increased optimal choice and decreased riskier choices at lowest dose
SCH 23390	0.001, 0.0032, 0.01, 0.032 (s.c.)	Koffarnus et al. (2011)	Male Sprague Dawley rats	↑				Decreased choice of large reinforcer at 0.01 and 0.032 mg/kg
	0.005, 0.01, 0.03	Simon et al. (2011)	Male Long Evans rats			–		No effect at any dose; risk associated with large reinforcer is mild footshock
	0.005, 0.01	St Onge and Floresco (2009)	Male Long Evans rats			↓		Both doses decreased risky choice, even when probabilities were high
	5, 10, 20 µg/kg	Wade et al. (2000)	Sprague-Dawley rats	–			
	0.001, 0.003, 0.01	Zeeb et al. (2009)	Male Long-Evans rats				–	Highest dose decreased number of trials and increased omissions

*↑ = increase in impulsivity, ↓ = decrease in impulsivity, – = no effect. Doses are expressed in mg/kg, unless otherwise specified. Route of administration is i.p., unless otherwise specified. Impulsive choice = Delay discounting (DD), Effort discounting (ED), Probability discounting (PD), and Rat Gambling Task (rGT)*.

Lending further support to the association between low DA and impulsive choice, numerous studies have found that laboratory animals choose smaller, immediate rewards more frequently following the administration of DA antagonists (see Table 5), including haloperidol (0.1–0.2 mg/kg, i.p.; 0.1 mg/kg, s.c.; Denk et al., 2005; Koffarnus et al., 2011), raclopride (80, 120 µg/kg, i.p.; Wade et al., 2000), flupenthixol (0.02, 0.03, 0.125, 0.5 mg/kg; 50, 100 µg/kg, i.p.; Cardinal et al., 2000; Wade et al., 2000), and SCH 23390 (0.02–0.03 mg/kg, i.p.; 0.01, 0.032 mg/kg, s.c.; van Gaalen et al., 2006a; Koffarnus et al., 2011). Very low doses of haloperidol (0.01, 0.03 mg/kg, s.c.), in comparison, had no effect on impulsive choice in response to standard delays, but decreased impulsive choice in response to delays that were much shorter than usual (Evenden and Ryan, 1996).

Dopamine antagonists, such as haloperidol (0.1–0.2 mg/kg, i.p.) and flupenthixol (0.25–0.5 mg/kp, i.p.) also increase effort discounting (Salamone et al., 1991; Denk et al., 2005; Floresco et al., 2008) and decrease the willingness to sustain effort as measured by progressive ratio breakpoints for natural rewards, such as food (Salamone et al., 2009) and pharmacological rewards, such as cocaine (Roberts et al., 2013). Interestingly, Cocker et al. (2012) reported evidence for differential effects of amphetamine on effort discounting in rats that exert high *vs*. low effort at baseline. In hard-working rats, a low dose (0.3 mg/kg, i.p.) increased their willingness to work to obtain the large reward, but higher doses (0.6, 1.0 mg/kg, i.p.) had the opposite effect. In the so-called “slacker” rats, these high doses enhanced their willingness to exert effort to obtain the large reward. These results are consistent with Aarts et al. (2014) findings of reduced cognitive control when DA levels are too high in individuals with greater DA synthesis capacity. It is possible that having too much DA impaired cognitive control in a way that made the smaller, but immediately available reward more appealing than the larger reward, which required greater effort to obtain.

Compared to temporal and effort discounting tasks, the results overall differ in probabilistic tasks where rats choose between a smaller but certain reward, and a larger but uncertain reward. In such tasks, low DA states induced by administration of flupenthixol (0.4 mg/kg, i.p.), eticlopride (0.01–0.03 mg/kg), and SCH 23390 (0.005–0.01 mg/kg, i.p.) decreased risky choices, even when probabilities of obtaining the larger reward were high (St Onge and Floresco, 2009; St Onge et al., 2010). In comparison to these effects of DA antagonists, the administration of small to moderate doses of amphetamine (0.125–1.0 mg/kg, i.p.) shifts choice preferences toward larger, more uncertain reward, even when the probability of delivery is very low (St Onge and Floresco, 2009; St Onge et al., 2010). This effect has been observed when such probabilities are presented in descending order, while the opposite was reported when probabilities increased over time (St Onge et al., 2010). The same research group has also found that low doses of amphetamine reduced delay (0.25 mg/kg) and effort discounting (0.125, 0.25 mg/kg, i.p.), but that a higher dose (0.5 mg/kg, i.p.) increased effort discounting, meaning that animals were less willing to exert effort to obtain larger rewards (Floresco et al., 2008).

The above findings suggest that distinct mechanisms underlie delay, effort, and risk discounting. They further highlight that methodological differences in task requirements or the order of presentation of various contingencies can be crucial when interpreting the effects of DA manipulations. Other differences, such as the presence of reward cues during the delay, the type of reinforcer used, and variations in the paradigms, are also worth considering. It is noteworthy that the standard delay discounting paradigm shares features with premature responding tasks such as the 5-CSRT, as both assess the ability to wait in order to get a reinforcer. This is supported by correlations between levels of delay discounting and premature responding in the same rats (Robinson et al., 2009). It is therefore possible that large increases in DA levels, which are known to induce premature responding, interfered with performance on DDT, and as such, masked the potential benefits of DA agonists on the ability to tolerate delays to maximize rewards. It thus appears that having too little or too much DA can impair performance on the delay and effort discounting tasks, whereas high DA might result in greater impulsivity on probabilistic discounting tasks when high probabilities are presented first.

On a recently developed rat version of the IGT, the rGT, *d*-amphetamine (0.25, 0.5, 1.0, 1.5, 2.5 mg/kg, i.p.) increased preference for smaller but certain rewards, resulting in poorer overall outcome (Zeeb et al., 2009; Baarendse et al., 2013; van Enkhuizen et al., 2013), whereas eticlopride (0.01 mg/kg, i.p.) improved performance by shifting preference toward larger but riskier rewards (Zeeb et al., 2009). It should be noted that rats were punished for losses by timeout periods during which no reward could be earned, which may indicate that amphetamine exerted its effects by increasing sensitivity to punishment. This interpretation is consistent with the observation that *d*-amphetamine (0.3–1.5 mg/kg, i.p.) dose-dependently decreased choice of a large but risky reinforcer in a probability discounting paradigm in which the risky reinforcer was associated with a mild footshock (Simon et al., 2009, 2011; Mitchell et al., 2011). Together, the above findings suggest that, in these studies, amphetamine affects risk aversion more clearly than reward sensitivity. There are challenges, though, in comparing rewards and punishments on features such as stimulus salience, intensity, etc. Other DA augmenters, such as cocaine (5–15 mg/kg, i.p.; Simon et al., 2009), GBR 12909 (2.5–28.5 mg/kg, i.p.; Baarendse et al., 2013; van Enkhuizen et al., 2013), and modafinil (16–64 mg/kg, i.p.; van Enkhuizen et al., 2013), as well as the DA antagonist SCH 23390 (0.001–0.03 mg/kg, i.p.; Zeeb et al., 2009; Simon et al., 2011), have not significantly affected performance on the rGT or on the probabilistic task with mild footshock.

#### Human studies

The DA—impulsive choice literature in healthy humans remains quite small (see Table 6). Most of the evidence—direct and indirect—suggests that low DA states aggravate impulsive choice while modest increases improve it (Trifilieff and Martinez, 2014). For example, transiently decreasing DA synthesis, using the tyrosine depletion method, impairs the ability to resist short-term, large gains despite long-term, larger losses on the IGT (Scarnà et al., 2005; Sevy et al., 2006), and decreases the willingness to sustain effort as measured by progressive ratio breakpoints when subjects work for alcohol (Barrett et al., 2008), tobacco (Venugopalan et al., 2011) and money (Cawley et al., 2013). On a guessing game in which probabilities of making the right decision vary, poor performance has also been observed following tyrosine depletion when probabilities were low (McLean et al., 2004), although a lack of effect of tyrosine depletion on probability discounting has also been reported (Lythe et al., 2005). Following administration of haloperidol (3 mg, p.o.), performance was impaired on a betting game in which there were no contingencies between responses and outcomes. Specifically, healthy controls who won money on a given trial subsequently increased the size of their bet on the next trial (Tremblay et al., 2011). Thus, greater reward expectancies resulted in increased risk-taking. However, the same dose of haloperidol did not affect performance on a slot machine game (Zack and Poulos, 2007), nor did a smaller dose (1.5 mg/kg, p.o.) affect rates of delay discounting in healthy adults (Pine et al., 2010). Pramipexole (0.5 mg, p.o.) increased risky choice on a gambling task following unexpected double wins (Riba et al., 2008). Again, reward expectancies influenced risk-taking. It thus seems that both high and low DA states enhance risk-taking when a large reward is expected.

**Table 6:** **Effects of altered DA transmission on impulsive choice in healthy human subjects**.

Drug	Dose	Study	Subjects tested	DD	ED	PD	IGT	Other gambling	Comments
**Increased DA transmission**
Amphetamine	20 mg	Acheson and de Wit (2008)	Healthy adults	–		–		
	10, 20 mg	de Wit et al. (2002)	Healthy adults	↓					Decreased discounting at 20 mg only
	10, 20 mg	Wardle et al. (2011)	Healthy adults		↓				Dose-dependent increase in willingness to exert effort to obtain larger rewards under low and medium probabilities
Bupropion	150, 300 mg	Acheson and de Wit (2008)	Healthy adults	–		–		
Pramipexole	0.25, 0.50 mg	Hamidovic et al. (2008)	Healthy adults	–		–		
	0.50 mg	Riba et al. (2008)	Healthy men					↑	Increased risky choice following “boosts” (i.e., unexpected double wins)
L-DOPA	150 mg	Pine et al. (2010)	Healthy adults	↑				
	100 mg	Symmonds et al. (2013)	Healthy adults					–	Lack of ongoing feedback during the task
**Decreased DA transmission**
Tyrosine depletion	90 g BCAA	Lythe et al. (2005)	Healthy adults	–		–		
	90 g BCAA	McLean et al. (2004)	Healthy adults					↑	Less likely to choose most likely option when probabilities were lower on a risk task
	62 g BCAA	Scarnà et al. (2005)	Healthy adults				↑		Variant of the IGT; Chose “experimental” gamble when losses were larger more often
	62 g BCAA	Sevy et al. (2006)	Healthy men				↑		Worse net scores over time
Haloperidol	1.5 mg	Pine et al. (2010)	Healthy adults	–				
	3 mg	Tremblay et al. (2011)	Healthy adults					↑	Correlation between payoff and subsequent bet size (no contingency between responses and outcomes)
	3 mg	Zack and Poulos (2007)	Healthy adults					–	No effect on slot machine game

*↑ = increase in impulsivity, ↓ = decrease in impulsivity, – = no effect. BCAA = branched chain amino acids. Impulsive choice = Delay discounting (DD), Effort discounting (ED), Probability discounting (PD), Iowa Gambling Task (IGT) and other gambling tasks (see text for details)*.

In comparison to these effects of decreasing DA transmission, healthy volunteers’ tolerance for delayed rewards on the DDT was increased by a moderate dose of oral *d*-amphetamine (20 mg; de Wit et al., 2002), but not following a lower dose of amphetamine (10 mg, p.o.; de Wit et al., 2002), 150 and 300 mg (p.o.) of the weak DA reuptake inhibitor bupropion (Acheson and de Wit, 2008), or the direct DA D2 agonist pramipexole (0.25–0.50 mg, p.o.) (Hamidovic et al., 2008). Low to moderate doses of *d*-amphetamine (10, 20 mg, p.o.) also decreased effort discounting, meaning that participants were more willing to work hard to obtain large rewards (Wardle et al., 2011). In contrast to the above findings, administration of the immediate DA precursor, L-DOPA (150 mg, p.o.), has been reported to increase delay discounting (Pine et al., 2010), while a smaller dose (100 mg, p.o.) had no effect on a gambling task in which no feedback was provided (Symmonds et al., 2013). It has been proposed that DA might affect decision-making through its effects on learning from different forms of feedback (Collins and Frank, 2014). The absence of ongoing feedback during the gambling task might have prevented L-DOPA from exerting effects, as no learning was involved. It remains unclear whether the conflicting results reviewed above reflect lack of specificity of some of the compounds used, different paradigms affecting different aspects of performance, different behavioral effects from changes in phasic *vs*. tonic DA release, spurious findings in a still small literature, or something else.

In summary, the evidence is less consistent when it comes to impulsive choice. Animal studies point to dose-dependent effects, with small increases in DA improving performance on the DDT and larger doses leading to impairment. In humans, decreasing DA transmission increases impulsive, effort discounting, but the effects of DA augmenters and behavioral responses on other tasks are less consistent. In studies using gambling paradigms, poorer performance is seen following elevated DA transmission in rats and lowered DA in healthy human subjects. Additional research in humans is needed where different drugs and a wide range of doses are directly compared.

## Summary and conclusions

It was previously proposed that increased *vs*. decreased DA transmission might predispose individuals to premature responding *vs*. delay discounting (Leyton, 2007). Since then, the animal literature has grown, and the proposed demarcation stands up well. Studies in neurologically intact humans, though, remain scarce, and caution is warranted since the exact effects in humans and rodents are not always the same. For now, it remains unclear whether these differences reflect methodology (e.g., different drugs, routes of administration and tests), neurobiology (e.g., larger, more complex and more dense DA innervation of primate frontal cortex), or, more simply, the smaller number of studies in healthy human subjects.

## Conflict of interest statement

The authors declare that the research was conducted in the absence of any commercial or financial relationships that could be construed as a potential conflict of interest.
